# The decentralisation-centralisation dilemma: recruitment and distribution of health workers in remote districts of Tanzania

**DOI:** 10.1186/1472-698X-9-9

**Published:** 2009-04-30

**Authors:** Michael A Munga, Nils Gunnar Songstad, Astrid Blystad, Ottar Mæstad

**Affiliations:** 1 Centre for International Health, University of Bergen, Bergen, Norway; 2 Department of Health Systems and Policy Research, National Institute for Medical Research (NIMR), Dar es Salaam, Tanzania; 3 Department of Public Health and Primary Health Care (ISF), University of Bergen, Bergen, Norway; 4 Chr Michelsen Institute, Bergen, Norway

## Abstract

**Background:**

The implementation of decentralisation reforms in the health sector of Tanzania started in the 1980s. These reforms were intended to relinquish substantial powers and resources to districts to improve the development of the health sector. Little is known about the impact of decentralisation on recruitment and distribution of health workers at the district level. Reported difficulties in recruiting health workers to remote districts led the Government of Tanzania to partly re-instate central recruitment of health workers in 2006. The effects of this policy change are not yet documented. This study highlights the experiences and challenges associated with decentralisation and the partial re-centralisation in relation to the recruitment and distribution of health workers.

**Methods:**

An exploratory qualitative study was conducted among informants recruited from five underserved, remote districts of mainland Tanzania. Additional informants were recruited from the central government, the NGO sector, international organisations and academia. A comparison of decentralised and the reinstated centralised systems was carried out in order to draw lessons necessary for improving recruitment, distribution and retention of health workers.

**Results:**

The study has shown that recruitment of health workers under a decentralised arrangement has not only been characterised by complex bureaucratic procedures, but by severe delays and sometimes failure to get the required health workers. The study also revealed that recruitment of highly skilled health workers under decentralised arrangements may be both very difficult and expensive. Decentralised recruitment was perceived to be more effective in improving retention of the lower cadre health workers within the districts. In contrast, the centralised arrangement was perceived to be more effective both in recruiting qualified staff and balancing their distribution across districts, but poor in ensuring the retention of employees.

**Conclusion:**

A combination of centralised and decentralised recruitment represents a promising hybrid form of health sector organisation in managing human resources by bringing the benefits of two worlds together. In order to ensure that the potential benefits of the two approaches are effectively integrated, careful balancing defining the local-central relationships in the management of human resources needs to be worked out.

## Background

Recruiting and retaining highly qualified health workers in remotely located areas presents an enormous challenge both in developed and developing countries. In both, the urban areas are generally perceived as more attractive because they are relatively more developed, and offer better working and living conditions to the workers, their families and relatives. Urban areas also seem to offer a wider range of economic opportunities because health workers can engage in private practice and other income generating activities to supplement their salaries. It is not surprising, therefore, for health workers to prefer working in urban areas. Remote places shoulder a huge number of public health problems and the associated challenge of few health workers per capita [1].

There have been a number of studies from both high and low income countries that attempt to analyse the most central factors for effective recruitment of health workers to remote places [1-4]. Most of these studies have focused on the attributes of particular locations where the challenge is greatest. Personal attributes of the prospective job-seekers or those who are already employed, such as rural origin, socio-economic, demographic and family background etc., have also been addressed. However, little emphasis has been placed on the potential role of the health sector organisation in recruitment. We shall argue that the hindrances and/or opportunities inherent in the health sector organisation (centralised or decentralised) may have substantial implications on recruitment, distribution and retention of health workers.

Our focus is on the implications of decentralisation vs. centralisation policies for the recruitment and distribution of health workers in remote districts of Tanzania. This country provides a unique case in the study of decentralisation vs. centralisation as the country has experienced major policy shifts in the recruitment of health workers, from centralised recruitment to decentralisation of recruitment procedures to a partial re-centralisation in the recruitment system in 2006.

Decentralisation has been defined as a process that involves one or all of the following aspects [5]: i) the shifting of workload from centrally located officials to staff or offices outside the national capital (*deconcentration*), ii) the transfer of management from the centre to semi-autonomous organisations and agencies within the public service structure (*delegation*), iii) the transfer of political and decision-making powers and authority for managing public services to independently elected local governments (*devolution*), and iv) the transfer of management and financing functions to a private organisation (*privatisation*).

The concept of decentralisation has become a catch word not only within health sector reform strategies but also in the overall policy and theoretical debates underpinning the development literature [6,7]. However, it is by no means a new concept. Decentralisation reforms gained a special status during the earliest primary health care reforms initiated by the World Health Organization (WHO) following the Alma-Ata Declaration of 1978 [8]. This declaration emphasised that community participation is a crucial ingredient for the development of responsive health-care systems both in high and low income countries. Community participation was seen as being achieved if more political powers and resources were relinquished from the centre to the local government authorities through a decentralisation process. Conceived in the framework of the primary health-care philosophy, decentralisation was supposed to ensure efficiency, effectiveness and accountability in the management of health-care services and resources [9,10].

There is a substantial body of literature on the decentralisation reforms in the health sector both from middle and low income countries. The literature largely centres on the impact the reforms have had on health system change and performance, with particular assessment of its influence on equity and efficiency in terms of financing and delivery of health services [11-15]. The reports have usually stated that, due to the lack of adequate and accurate empirical data, it is difficult to reach firm conclusions on the extent to which health sector decentralisation reforms shape health system change and performance. Moreover, such studies have brought up the importance of contextual factors in shaping the processes and outcomes of the reforms [6,11,12,16,17].

A few studies carried out in Tanzania have focused on identifying the type of changes that decentralisation policies have brought about for the management of resources, service provision, and accountability and good governance [18-20]. The results highlight both positive and negative effects. On the positive side, aspects such as the increased authority of the district councils to spend user fees to repair health facility infrastructure and purchase drugs has increased the flexibility in planning for health services at the district level. In addition, increased local accountability is another aspect that has been consistently brought up. On the negative side, the challenges related to unclear and complex local-central relations in terms of managing health sector resources has been highlighted. Little emphasis has been placed on assessing the impact of decentralisation reform on health worker recruitment, and its implications for the distribution of health workers across districts in the country. Evidence from other low and middle income countries suggests that decentralisation reforms may have unintended negative implications on the employment structure [21]. Decentralisation reforms seem to have created or heightened internal labour market competition and development of new avenues for the uneven distribution of health workers across local administrative units. This has led to an aggravation of pre-reform inequalities in the distribution of health workers [21-23].

We present the findings from an exploratory qualitative study of the impact of the shifting centralisation-decentralisation reforms on health worker recruitment in Tanzania. In particular, the opportunities and challenges experienced by decentralised recruitment systems are discussed, as well as by the recent partly reinstated central recruitment system. The recent policy shift gives us a timely opportunity to investigate the benefits and bottlenecks experienced in both recruitment systems. We argue that there is a need to work for a fine-tuned balance between decentralised and centralised management of health worker recruitment. Ultimately, we aim to contribute further knowledge that can prevent quality health services from being primarily an offer to the affluent urban dwellers.

The following section sets out the context by focusing on policy developments regarding the organisation of the recruitment of health workers in Tanzania. The research questions and methodology are presented in the subsequent sections. In the final three sections, we present and discuss the results and summarise the main conclusions and policy implications.

## The context

### Tanzania's socio-economic indicators and health system structure

The country is divided into mainland Tanzania and the Zanzibar islands and has a population of 37.4 million [24]. Tanzania is one of many sub-Saharan African countries with severe poverty. The average life expectancy at birth is 45.6 years [24,25]. The latest Tanzania Demographic and Health Survey [25] revealed poor health indicators with infant and under-five mortality estimated at 68 and 112 per 1,000 live births, respectively. Maternal mortality is 578 deaths per 100,000 live births, with less than half (43%) of all births in the country being attended by skilled attendants [25]. These figures are comparable to the other East African countries.

The health system in Tanzania comprises the public, the private for profit, and the voluntary agency sectors. The public health system is organised in a referral structure that has a pyramidal form. The lowest level consists of health posts and dispensaries. Above the dispensary level, there are health centres and district hospitals. In districts without a district hospital owned by the government, voluntary agency hospitals will serve as Designated District Hospitals (DDH). Above the district level are the regional hospitals. There are four tertiary hospitals at the highest level.

The health worker census of the Ministry of Health and Social Welfare (MoHSW) states that Tanzania has a total of 48,508 health workers [26]. The country has 0.02 physicians per 1,000 inhabitants, the lowest in the world [27] The public sector employs 70% of the health workforce [28], and about 40% of the health workers are unskilled. A recent analysis indicates huge inequalities in the distribution of health workers between rural and urban districts, with a range from 0.3 health workers per 1,000 inhabitants in one rural district to 12.3 health workers per 1,000 inhabitants in one urban district [28].

### The centralised phase: pre-1982

Tanzania has a unique post-colonial history of early implementation of socialist reform policies following the Arusha Declaration of 1967. The period spanning from the colonial period through Tanzanian independence to 1982 was characterised by a strong centralisation and parallel lack of a local government system in its real sense. All production and distribution of economic and social services, such as health and education, were centrally managed. The socialist reforms implemented following the Arusha Declaration were meant to, among other things, reorganise the government administration to facilitate the implementation of the post-independence policies, such as massive investment in education and health infrastructure, and the creation of village cooperative farms [29].

The implementation of social policies under the auspices of the Arusha Declaration created expectations from both policy makers and the population that social services would be extensive and effectively reach the whole population. However, the effectiveness of socialist reforms was limited, and the intention in the Arusha Declaration policies to decentralise power and authority to local communities were impossible following a 1972 parliamentary act [30]. The 1972 act centralised power and authority even further to the government, despite some degree of deconcentration of tasks from the centre to the regions. In effect, the existing (albeit weak) local governments at the district level were removed by the 1972 act. From 1972 onwards, all matters related to the management of public servants, including the recruitment and distribution of health workers, were controlled by the central establishment office, known since the early 1990s as the Civil Service Department (CSD).

In the health sector, one of the main challenges was soon experienced to be the recruitment and distribution of health workers. A serious mismatch between local needs for human resources and the number of health workers allocated to the districts by the central government was among the main problems that were expected to be addressed when Tanzania turned towards a more decentralised structure of government.

### The decentralisation phase from 1982

During the early 1980s, Tanzania prepared the ground work for major decentralisation reforms. Six important pieces of legislations were passed in 1982 [31-36]: a) the Local Government (District Authorities) Act Number 7 of 1982, b) the Local Government (Urban Authorities) Act Number 8 of 1982, c) the Local Government Finances Act Number 9 of 1982, d) the Local Government Services Act Number 10 of 1982, e) the Local Government Negotiating Machinery Act Number 11 of 1982, and f) the Decentralisation of Government Administration (Interim Provisions) Act Number 12 of 1982. These parliamentary acts aimed to institute the local government with democratic structures and institutions that had been paralysed by the 1972 Parliamentary Act [30]. Decentralisation gained further momentum from the mid 1990s and onwards within the framework of the Local Government Reform Programme (LGRP) that was charged, among other things, to ensure that districts have relevant and capable structures of governance to manage their own affairs. Under this arrangement, management of staff was expected to be decentralised so that local government authorities could appoint, develop and discipline their own staff [18,37].

To prevent unnecessary political interference in the recruitment processes at the district level, the Public Service Act of 2002 and the Public Service Regulations of 2003, inter alia, reduced the number of district council members needed to form the Employment Board (currently there is only one member of the district council in the board). From 1982 to 2002, the Finance and Planning Committee, largely composed of local politicians, had been the main body charged with the management of recruitment at the district level. By strengthening the role of technocrats in matters related to the recruitment of workers at district level, unnecessary political interventions in matters related to recruitment of workers were expected to be reduced.

While the law provides the mandate to districts to manage the recruitment of health workers, several central government departments continue to have key roles in the management of workers at the local government levels: a) the Ministry of Finance approves districts' budgets and sets guidelines for the spending of locally mobilised financial resources as well as central government allocations, b) the Civil Service Department has a central role in approving employment permits and, in collaboration with the Public Service Commission, confirms health workers' employment and manages their promotion, and c) the Regional Administration and Local Government ministry (currently under the Prime Minister's Office) approves health personnel transfers from one district to another.

Practically, the recruitment process in the context of decentralisation is supposed to take place hand-in-hand with the budget process both at the local and central government levels. The estimates indicating the numbers and types of workers, and the associated costs (personnel emoluments), are discussed and recommended by all the local governments' committees before they are tabled at the full District Council for endorsement. After agreeing upon the number and type of workers (e.g. cadres of health workers) to be recruited in a particular financial year, the budget estimates are presented to the Civil Service Department for approval and processing of employment permits. At this stage, the Civil Service Department either endorses the estimates or adjusts them depending on what has been centrally approved by the Ministry of Finance in the budget for the particular ministry. This leads to an employment permit being issued, and the respective district authority is informed so that vacancies can be announced. The district authority is required to act on the issued permit within a three month deadline, after which it expires. After the information has been channelled to the district authorities, the District Executive Director (DED) informs the district Employment Board and the Public Service Commission of the existence of a funded vacancy in the respective district. (Note that, the Employment Board consists of a chairperson who is a respected person in the respective district, one district council member, a District Administrative Secretary or his/her representative, a Local Government Officer from the Regional Secretariat and a representative from the Public Service Commission). Upon communication with the Employment Board, the DED instructs the district's Human Resources Officer to advertise the vacant posts in the local and national media. After receiving sufficient responses to the advertisement, the DED through the District Human Resources Officer prepares a shortlist of applicants with required qualifications for the advertised job. The Employment Board sets up a panel to interview the short-listed candidates. At this point, the names of the best candidates at interview are taken to the District Planning and Finance Committee for endorsement, and finally the candidates are offered letters of appointment, with copies to the Public Service Commission, the Civil Service Department and the Ministry of Finance for information.

### Partial re-centralisation in the context of decentralisation: from 2006

Responding to a series of reported serious problems related to the recruitment of health workers, teachers and accountants under the decentralised arrangement, the central government decided to reinstate in part a centralised recruitment system of health workers, teachers and accountants through the issuance of a Presidential Establishment Circular (BC. 46/97/03/A/123) in November 2006. With this new circular, the government hoped to improve the distribution across the country of health professionals available in the labour market and those fresh from training institutions. During the 'old' centralised phase, districts would receive whatever was posted from the central government. The new system of partial centralisation provides more room for district authorities to plan what they need in terms of numbers and types of health workers, and to submit their requests to the central level. Based on these needs, the Ministry of Health and Social Welfare, the Public Service Commission in collaboration with the Civil Service Department liaise with the training institutions to find candidates to fill the identified gaps. In addition, vacancy announcements are made by central authorities to target those already in the labour market. All the arrangements for short-listing and interviewing the best candidates are also centrally managed before candidates are posted to respective districts. We emphasise that this return to 'centralised' recruitment procedures is by no means a full retreat to a pre-decentralisation recruitment mechanisms. The districts have an upper hand in some crucial processes, such as in identifying the requirements for health workers and managing the budget processes at the local level. They also have a final say in accepting or rejecting the posted candidates.

### Research questions

Decentralisation reforms are expected to make the government more responsive to local needs through better utilisation of local information and stronger accountability systems. Delays in planning and executing plans may be reduced through less overload and congestion in the channels of administration and communication, and through simpler bureaucratic procedures. Ultimately, the quantity and quality of public services may improve [9,17,21,37].

We investigated how these and other mechanisms have unfolded in the specific context of recruitment of health workers in Tanzania. Firstly, we asked how decentralisation has affected the responsiveness to local needs and the accountability systems in relation to the recruitment of health workers. Secondly, we explored whether decentralisation leads to less bureaucracy and more efficient processes of health worker recruitment. Thirdly, we focused on the possibility that decentralisation heightens the competition between districts for qualified workers, and how the more disadvantaged districts in Tanzania fared in this competition.

## Methods

### Study design

We conducted a qualitative exploratory study between August and September 2007. It was carried out at district and national levels in Tanzania. Five districts of mainland Tanzania were included, namely Ngara District (Kagera Region), Meatu, Bukombe and Bariadi Districts (Shinyanga Region) and Kongwa District (Dodoma Region). These are the districts with the lowest number of health workers per capita, according to the 2001/2002 Human Resources for Health Survey from the Ministry of Health and Social Welfare [26] and the 2002 Population and Housing Census [38].

### Key informant interviews

In total, 21 interviews were carried out. In each of the five districts, the District Medical Officer (DMO), the District Human Resources Officer (DHRO) and the District Executive Director (DED) were interviewed. The informants were chosen purposefully to benefit from their knowledge based on their strategic positions in human resource management and health workforce planning. Fifteen in-depth interviews were conducted at district level. The national level informants were recruited from the central government, the NGO sector, academia, and foreign development partners with a stake in the health sector. Six interviews were conducted at national level.

The initial plan was to get 18 informants from six districts, and a total of 10 informants were planned to be recruited at the national level. There was, however, evidence of repetition of major emerging themes after completing the interviews in five districts and interviews with the national level informants, which indicated some "data saturation". It was perceived that sufficient comprehensiveness of responses and depth of knowledge had been ensured to discontinue the inclusion of new informants.

An interview guide with open-ended questions was employed during the interviews. New and important issues that emerged in the course of the interviews were added to this guide and were further explored in the subsequent interviews. All the interviews were carried out face to face in Swahili, the official language in Tanzania, except one interview with an informant from an international organisation. Both audio-taping and note-taking were used to record the information.

### Data analysis

Thematic content analysis was used on the data in an iterative manner. Tape-recorded interviews were transcribed word by word. Initial familiarisation with the data through repeated review of transcripts and field-notes was done at this stage. We employed multiple-coding, i.e., coding and interpretation of codes were carried out by different researchers [39]. Three researchers were involved in this process. This was done in attempts to create codes and categories that as closely as possible reflected the content of the data. Concepts used by the informants rather than the questions raised in the interview guide were employed as codes [39]. The coding categories extracted from the transcripts were used to analyse systematically topics that were repeatedly mentioned in making up patterns of experience. Apparent contradictions emerging from the material were singled out to allow for the comparison of the information given by different informants.

### Ethical considerations

Ethical clearance to conduct the study was obtained from the National Institute for Medical Research (NIMR), Tanzania. Verbal consent was sought from prospective interviewees after explaining the aims and methods of the study, subsequently informing them of the research ethical principles of voluntary participation, the right of withdrawal, and the principles of anonymity and confidentiality.

## Results

The results present the informants' experiences with the decentralisation and the re-instated centralisation of health worker recruitment. Before turning to these issues, we start by briefly commenting on informants' general experiences with the decentralisation reforms in the management of the district health services.

The implementation of decentralisation reforms was commended by most of the informants in terms of increased local participation in identifying needs for health care delivery and financing. Decentralisation was also praised for reducing the problem of mismatch between what the districts actually need and that made available by central government. The informants indicated that local priorities related to social development sectors such as health, education and water were to be reflected in the consolidated district budget. In addition, the creation of District Health Boards (DHB) and Council Health Management Teams (CHMT) helped to improve the management of health services. For example, under the decentralised arrangement, the DHB in consultation with the CHMT and with the approval of the District Council facilitated the use of available funds in ways that had not been possible under centralised government. In this case, decentralisation had increased the flexibility in local planning and ownership of health projects at the district level.

### Decentralisation and recruitment of health workers

#### Planning according to need

It was consistently learnt that the recruitment process under the decentralised arrangement was closely linked to the budget process both at the local and at the central level. During the process of estimating personnel emolument requirements, the District Executive Director's office was reported to work in close collaboration with the District Medical Officer (DMO) to prepare the health worker requirements for all health facilities in the district. The officer-in-charge at each health facility in the district submitted the health personnel requirements to the DMO's office. Supervisory visits completed by CHMT members to health facilities provided another source of information that identified the specific need for health workers.

The implementation of the decentralisation policy was credited by a majority of the study informants as providing more room for district authorities to request what they needed compared to the recruitment under centralisation, whereby the central government would post health workers to district without taking into consideration the specific needs of each district. Thus districts requiring more health workers could very well end up receiving employees for other sectors instead.

#### The influence of local politics and patronage

While there was broad consensus that decentralised recruitment of health workers is responsive to specific local needs, some of the informants pointed out that some influential local politicians in the District Council interfered with the recruitment process. There were reported cases of District Council members pushing for the recruitment of categories of civil servants in line with their relatives' qualifications, even when the district did not need them. As one informant at district level indicated:

"Some three to four years ago, I remember we requested nurses and clinical officers to fill the gaps existing in our health facilities, but the council had changed the budget to look as if we needed more agricultural extension officers while we actually demanded for health workers ... and it has later on been learnt that some councillors had their relatives who qualified as Agricultural Extension Officers whom they wanted to be assured of employment."

In three districts where patronage in the conduct of local politics was rife there were reports that the quality of the district workforce was compromised by employment of unqualified workers because of undue influence on the recruitment process by district officials. Informants cited instances of threats against health managers, such as the District Medical Officer, if they did not comply with the demands of the local politicians. This finding indicates that the decentralised recruitment opened new avenues for political influences on technical decisions related to employment of health workers.

#### Complex and costly recruitment procedures

It was conceded by a majority of informants that the recruitment procedures are complex and time-consuming, and several informants referred to attempts at recruitment that had failed. It was also explained that the time taken from the request for the permit to the actual recruitment was approximately one year. A recruitment permit has a duration of only three months, and in many cases, the informants claimed that the districts failed to recruit the required health workers within the deadline. The reason given was lack of responses from qualified applicants, even after several advertisements of the vacant posts. In particular, this was seen as a problem when a vacant position required a highly skilled health worker. In general, the complex bureaucratic procedures under the decentralised arrangement were considered difficult and expensive to manage, especially when the process needed to be repeated due to failure to recruit in the first place, or because the recruited candidates did not show up. One informant noted that:

"You can get the qualified one, interview him and offer him a letter of employment but he does not show up. Hence, you need to restart the process of advertising, inviting applicants, and convening interview panels, which all are costly."

The majority of informants raised the concern that the process of recruitment under the decentralisation system with its inherent bureaucratic procedures, such as permit processing by the central government, had contributed not only to delays in recruiting health workers, but also to increased costs of managing the process in the poorer remote districts. It was suggested that in order to improve recruitment under a decentralised setting, the Civil Service Department should not attach stringent deadlines to the recruitment permits.

#### Retention of health workers

The decentralised recruitment arrangement was praised as being effective in terms of retaining the recruited health workers, especially when the employees were recruited from within the local or nearby districts. These individuals would be fully aware of the working and living environment where they would start working, and therefore would not be surprised by the lack of services. It was also conceded by a majority of the informants that employees posted by the central government would not have the same commitment to work in a rural district as those who had applied directly to the district authorities. As an example, one informant stated:

*"We may be sure that the job seekers have done a thorough assessment of the work and living conditions of the place they are about to go and work and convince themselves that they will cope*."

Other informants, especially at the national level, argued that it is difficult to assess directly whether one recruitment system is better than the other in relation to retention of workers.

In principle, decentralised recruitment may improve the retention of both highly skilled health workers and lower cadres, because employers can match the expectations of prospective health workers to the actual living and working environment. However, a view shared by the majority of informants was that qualified people, especially highly skilled workers, were difficult both to recruit and retain because they are in high demand in urban areas and the private sector.

Another challenge mentioned was that health workers, especially those who had an urban background, would use the remote districts job vacancies as a bridge to getting government employment in urban areas. This implies a higher turnover and added costs of recruitment for the remote districts. One informant pointed out that:

"Because living and working conditions in the villages are not conducive, health workers, especially women, when they are posted here they quickly start to make marriage arrangements and secure marriage certificates with a husband who lives in places such as Dar es Salaam. We are not sure of the social and moral commitments surrounding these marriages. Soon they start processing transfers for reasons of family re-union. If the request is approved, the worker then leaves our district. So in some cases, unattractive places, such as our district, are used as 'stepping stones' to get employment in the public service."

#### Responsibility without authority

A thematic pattern that consistently emerged in the interviews was that districts are being assigned too many responsibilities that do not match with the resources at their disposal, a phenomenon described as '*responsibilities without resources and authority*". In relation to health personnel, there was a consistent concern that the authority to manage health personnel issues is constantly overridden by a number of central government organs with a stake in the management of public servants. This, they pointed out, leaves very little room for the district authorities to have a say in the management of their health workers and seriously reduces the effectiveness of the decentralised recruitment, retention and distribution of workers across districts.

Most of the informants held the view that in order for 'decentralisation's good intentions' to be realised, excessive and unnecessary central government interventions on matters of otherwise local nature need to be removed. They pointed out that the local authorities' limited power and authority in decisions on matters such as using locally mobilised finances to pay and motivate their health workers is one of the key impediments to attracting and retaining the required workers. If financial regulations are made more flexible, there is a potential that health managers at the local level can devise innovative ways of attracting workers. One informant at the national level provided this example of a local initiative in recruiting health workers:

"There was one in-charge working with a District Designated Hospitals in one region who once came to the Ministry of Health to request the assistance of the ministry in getting medical doctors for their hospital. The ministry could not give the assistance required. The in-charge went to Bugando University College of Health Sciences in Mwanza Region and talked directly to the doctors. She told them the benefits they would get by working with their hospital in terms of both financial and non-financial incentives. She finally succeeded to get four doctors who agreed to go to that region after their graduation. If all district councils were given the flexibility to directly spend some of their resources to attract workers, many problems of shortage in hard-to-staff districts would have been solved."

#### Distribution of health workers

Whilst the decentralised recruitment was praised as being effective in recruiting and retaining health workers from within the local and nearby districts, decentralisation was however perceived to have aggravated pre-reform imbalances in health worker distribution between the districts. It was argued that the recruitment system had exacerbated the competition for health workers among the districts, and between local and central government employers. The remote districts come out as losers in this process. It was learnt from informants that the failure of remote districts to recruit was due to costly and complex procedures surrounding the decentralised recruitment process, combined with the relatively small financial resources and weak institutional capacity of these districts.

### Centralised recruitment in a decentralised health sector

As described above, since 2006 the central government has intervened in the recruitment of teachers, accountants and health workers. Many informants described the reinstated central recruitment as being 'operationally complimentary' to the decentralisation reforms, because it has helped districts to get more highly skilled health workers in a relatively easy way compared to the previous, decentralised arrangement. Furthermore, the new system seems to have reduced the cost of the recruitment process related to advertisements, interviewing etc., which would otherwise be shouldered by local governments.

Compared to the decentralised system of recruitment, the re-instated central recruitment of health workers was perceived by a large majority of the study participants to be more effective in balancing health worker distribution across districts as it has additional mechanisms for ensuring that health workers are distributed relative to the needs of the districts. However, informants also explained that some of those who are deployed to rural districts fail to turn up, e.g. for reasons related to employment in the private health sector or for personal reasons. One informant at district level remarked:

"In the financial year 2006/2007 the central government has posted five clinical officers to come to work in our district, but it was only two who reported and stayed, and the Ministry of Health and Social Welfare did not do any follow-up to see whether the posted health workers reported or not. We had several times tried to communicate this problem of non-reporting to the ministry, but nothing has so far occurred."

A majority of the informants did believe that there was a need for a division of responsibilities between the districts and the central government in matters related to recruitment and distribution of health workers. Highly skilled health workers, such as medical officers, dentists, radiographers and laboratory technicians, are hard to find in remote rural districts, and informants believed it is important that the central government manages the whole process of recruitment and distribution of these particular categories of health workers. The recruitment of unskilled health workers, on the other hand, was said to be very well managed by the decentralised districts because these categories of health workers could easily be obtained within the district or from nearby districts.

## Discussion

Whilst the existing literature on the impact of decentralisation in the health sector has largely focused on resource management in general and its impact on health system performance [11-13,16-18,37], our research highlights the ways in which decentralisation may affect the recruitment and distribution of health personnel. We have also attempted to add to the present knowledge-base by juxtaposing the different historic periods in Tanzania, contrasting decentralised recruitment with a more centralised recruitment process.

The results have indicated that, while decentralised recruitment provides opportunities for a more responsive planning of health workers as it is sensitive to local needs, three key issues continue to be stumbling blocks for effective and efficient recruitment of health workers to remote districts. The first relates to the limited power and authority vested to district authorities to manage complex health personnel issues. This challenge was referred to by our informants as 'responsibilities without authority'. One explanation of this challenge is that decentralisation in Tanzania, not the least that part pertaining to the management of health workers, can at best be characterised as a *partial *decentralisation. Informants reported unnecessary interference from a number of central government organs in the management of human resources. Braathen et al. [18] compared Tanzania with Uganda and concluded that Tanzania is lagging behind Uganda in terms of the amount of autonomy the central government had relinquished to the local governments. In a study of bottlenecks in the recruitment process in Tanzania, Martineau [19] identified complex, long and bureaucratic personnel deployment processes as one reason for delays and sometimes failure to recruit health workers in hard-to-staff parts of the country. A report by Blunder [40] presented similar observations.

Some informants argued that increased local autonomy to spend locally mobilised resources would reduce the problem of recruiting health workers in remote districts, because this would empower districts to design incentive mechanisms that could attract the workers. While this might be the case if only the remote districts got this opportunity, it is more doubtful that the same would be the case if increased autonomy were extended to all districts. As long as districts compete for scarce health workers, resource deprived districts are likely to lose out.

Second, decentralisation in Tanzania took place in the midst of weak local institutions that could easily be manipulated by local elites to marshal their personal interests. Local politicians were initially given wide powers to manage the recruitment process at the district level. The legal powers of local politicians in the recruitment process were reduced by the Public Service Act number 8 of 2002 [41] and its corresponding Public Service Regulations of 2003 [42]. However, our results indicate that some local politicians remain influential in the recruitment process through more informal mechanisms, including threats.

The third stumbling block relates to bureaucratic constraints, such as the long process of requesting employment permits necessary for recruitment of health workers. According to the informants, this causes both delays in the recruitment and additional costs, especially in the remote areas where the recruitment process often has to be repeated due to the short duration of employment permits, failure of recruited candidates to show up, and the generally high turnover rate of skilled health workers.

Some of the identified problems related to recruitment of health workers may well be attributed to the fact that the decentralisation reforms in Tanzania were implemented without adequate preparations. Kolehmainen-Aitken [43] has pointed out that in many countries, managers who were supposed to implement decentralisation reforms were not only untrained and ill-prepared, but were also working in a context of weak personnel systems. One reason for lack of adequate preparations may be opposition from bureaucrats in the central administration. Gilson and Mills [16] have indicated that, during the initiation and design phase of the decentralisation policy in Tanzania and Papua New Guinea, the opposition from centrally positioned bureaucrats undermined the decentralisation process due to fears of losing power and prestige; they allowed weak governance structures to strengthen the role of the centre instead of enhancing effective local government structures. For instance, no mechanisms were put in place to help the perceived unattractive employers to recruit qualified (health) workers. Youlong et al. [22] have similarly argued that, amidst stiff competition for health workers as a result of implementing decentralisation reforms in China, there were no effective mechanisms for attracting and retaining health workers in remote areas. Similar concerns were raised by our informants.

The opposition by bureaucrats to fully implement the reform policies may have been fuelled by the fact that decentralisation reforms were strongly pushed by foreign donors. Wang et al. [21] and Werlin [44] have argued that the driving forces for decentralisation in the health sector of most low and middle income countries did not always come from the health sector itself. In addition, the early reformers often provided little room for consultations with the health sector stakeholders [13,21]. Bossert and Beauvais [13] have indicated that the Ministry of Health at the Philippines was inadequately consulted at the time of the design and initiation of the decentralisation reforms, a phenomenon that led to a number of implementation problems related (but not limited) to the management of human resources. In Tanzania, the implementation of decentralisation reform was carried out when the government was responding to the pressure from international financial institutions such as the World Bank, and the reform was tied by the aid conditionality from the donors.

Our results further indicate that centralised recruitment may be more effective in both recruiting highly skilled health workers and distributing them across districts relative to the reported needs, although doubts were reported of the effectiveness of the central recruitment system in ensuring that the recruited health workers stay in the district to which they are posted. This somewhat rosy picture painted by health managers of the 'goodness' of centralised recruitment needs to be interpreted with some caution. First, it might be the health managers' confession of despair after their failure to attract and retain health workers in their districts, whereas the real problem is that these districts are simply not attractive enough to prospective job seekers. Second, the positive attitudes towards more centralised recruitment may be due to the partial nature of the decentralisation reforms which, as evidenced by our findings, had left health managers with little power and authority to effectively manage the recruitment and retention of health workers. The partial recentralisation may thus be seen as a way to alleviate the problems caused by the partial decentralisation.

### Weakness of the study

One weakness of our study might be that we consulted only the most poorly staffed districts. There may exist relatively resource poor districts that have been relatively more successful in attracting health workers under the decentralised system. Their experiences might have shed further light on the importance of the local endowments of resources for the ability to attract workers. Furthermore, the informants' views about the counterfactuals – for instance, what would have happened if districts were entrusted with even greater local autonomy – should be interpreted with caution.

Except for differences in views about the impact of decentralisation on the retention of health workers, this study found little disagreement amongst informants about the issues that were discussed. This could be strength, but it could also be an indication that the group of recruited informants was too homogeneous.

## Conclusion

Decentralisation reforms in Tanzania have resulted in more effective utilisation of local information in the recruitment of health workers, ensuring better planning according to need. However, bureaucracy and delays in the recruitment processes remain important problems.

Moreover, decentralisation reforms have created new forms of competition for scarce human resources, including health workers. Such competition may increase pre-reform imbalances in the distribution of health workers, especially when the recruitment process is complex and costly and poorly staffed districts have less financial resources and a weak institutional capacity compared to other districts. In order to prevent or mitigate such outcomes, a 'hybrid' form of recruitment where the central government plays a role as 'facilitator' could be an optimal solution.

An alternative to the hybrid model would be to more directly address the underlying problems to which the hybrid form is responding, by a) reducing the cost of the recruitment process to give greater autonomy to the decentralised districts, and b) endowing poorer districts with added financial resources and greater institutional capacity. To add to the effect of b), districts can also be given increased financial autonomy so that they can be flexible enough to devise innovative ways of attracting and retaining health workers. One such innovation, mentioned by our informants, would be to recruit health workers on short term contracts by using locally mobilised financial resources to cover salary costs while waiting for the result of the normal, long and bureaucratic process of recruiting workers on a permanent basis.

We should emphasise that the optimal way to organise the recruitment of health workers will strongly depend on contextual factors. Further research will be needed to identify how to optimally balance the involvement of the central government against the autonomy of local authorities in each particular setting.

Finally, the organisation of recruitment process is, of course, only one of the factors that affect the distribution of health workers. In addition to effective recruitment procedures, special incentive packages may also be needed in order to ensure a more equitable distribution of health workers in low income settings, especially where the local districts differ greatly in terms of the living conditions they are able to offer to prospective job seekers.

## Competing interests

The authors declare that they have no competing interests.

## Authors' contributions

MAM conceived the study, collected the data, analysed the data, prepared the first drafts and participated in all subsequent processes of manuscript development to its publishable form. NGS participated in the design of data collection tool, data analysis and preparation of the manuscript. AB contributed to the conception of the study, participated in the preparation of data collection tool, data analysis and manuscript development. OM conceived the study, participated in the study design and development of the data collection tool and critically reviewed all drafts of the manuscript. All authors have read and approved the manuscript to be submitted as it is.

## Pre-publication history

The pre-publication history for this paper can be accessed here:

http://www.biomedcentral.com/1472-698X/9/9/prepub
